# Development of a Vitality Scan related to workers’ sustainable employability: a study assessing its internal consistency and construct validity

**DOI:** 10.1186/s12889-015-1894-z

**Published:** 2015-06-16

**Authors:** Livia AM Brouwers, Josephine A Engels, Yvonne F Heerkens, Allard J van der Beek

**Affiliations:** Research Group Occupation and Health, HAN University of Applied Sciences, P.O. Box 6960, 6503 GL Nijmegen, The Netherlands; Department of Public and Occupational Health, EMGO+ Institute for Health and Care Research, VU University Medical Center, Amsterdam, The Netherlands

**Keywords:** Instrument, Measures, Stagnation, Clinimetrics, Sustainable employability

## Abstract

**Background:**

Most validated sustainable employability questionnaires are extensive and difficult to obtain. Our objective was to develop a usable and valid tool, a Vitality Scan, to determine possible signs of stagnation in one’s functioning related to sustainable employability and to establish the instrument’s internal consistency and construct validity.

**Methods:**

A literature review was performed and expert input was obtained to develop an online survey of 31 items. A sample of 1722 Dutch employees was recruited. Internal consistency was assessed by Cronbach’s alpha. The underlying theoretical concepts were extracted by factor analysis using a principal component method. For construct validity, a priori hypotheses were defined for expected differences between known subgroups: 1) older workers would report more stagnation than younger workers, and 2) less educated workers would report more problems than the highly educated ones. Both hypotheses were statistically tested using ANOVA.

**Results:**

Internal consistency measures and factor analysis resulted in five subscales with acceptable to good reliability (Cronbach’s alpha 0.72-0.87). These subscales included: balance and competence, motivation and involvement, resilience, mental and physical health, and social support at work. Three items were removed following these analyses. In accordance with our a priori hypothesis 1, the ANOVA showed that older workers reported the most problems, while younger workers reported the least problems. However, hypothesis 2 was not confirmed: no significant differences were found for education level.

**Conclusions:**

The developed Vitality Scan - with the 28 remaining items - showed good measurement properties. It is applicable as a user-friendly, evaluative instrument for worker’s sustainable employability. The scan’s value for determining whether or not the employee is at risk for a decrease in functioning during present and future work, should be further tested.

## Background

In today’s Western world, people have to work longer before they can retire and the working population is aging [1–4]. Employees are confronted with a fast changing work environment and continuously increasing work demands [5]. Therefore, the need is growing for a sustainable career perspective and so-called life-long learning to face these challenges [6, 7].

Sustainable employability can be defined as an employee’s capability to participate in present and future jobs while preserving good health and well-being as well as the necessary conditions for this to occur. Whereas research on healthy careers merely focuses on an employee’s mental and physical state, sustainable employability requires a supportive work environment and employees with the attitude and motivation to explore and develop their capabilities [8]. For employees it is important to stay healthy in and engaged at their jobs. This requires attention for personal and work related factors. Sustainable employability is vital for the economy and for employees themselves. Both employers and employees must cope with fluctuating demands on flexibility and changing job requirements, now and in the future [8–10].

Hence, sustainable employability is not a personal characteristic, but rather the result of an interaction between the individual and their work [11]. A good balance between the strain (physical and mental) caused in the job and the employee’s working capacity is crucial to keep employees healthy and vital at work [11–14]. In monitoring sustainable employability, it is important to detect signs of stagnation in functioning as early as possible [14]. As Van den Tooren *et al.* [15] have pointed out people can compensate for their high job demands and prevent stagnation, through self-regulation processes, such as activating supportive job resources or mentally detaching from work when at home. Taking note of an employee’s declining compensation as early as possible may contribute to prevent stagnation. A match between person and (work) environment is the result of a wide range of interdependent factors. The employee brings in personality as well as emotions and motivations, where the job is a combination of specific tasks along with cultural aspects and development possibilities. Since a good fit between an employee and his or her job is determined by the work context as well as personal characteristics, a combined effort is needed from both employee and employer to attain and maintain this *person-job fit* [11]. Employees are a heterogeneous group with diverse personality traits, needs, competences and ambitions [16]. Their health and well-being depend on these personal characteristics as well as on work related environmental factors such as work and task demands [17].

Often employees who experience difficulties in their sustainable employability – whether or not these difficulties are caused by work-related or other factors – do not feel secure enough to discuss the issues, e.g. poor health, a decline in motivation or difficulty in keeping up with their work, with their employer or supervisor. Employees may feel vulnerable when admitting to their employer or supervisor that their performance might be at stake. On the other hand, employers and supervisors can be insecure about how and when to best discuss such issues with their employee [18]. Opinions differ as to the extent to which this is in fact something an employer is allowed to address. The dividing line between private matters or business-related problems is often indistinct. Instruments assessing the employee’s vitality, work ability, and individual development might be helpful for starting a dialogue between the employee and employer or supervisor based on objective information.

Conceptualizations of the dimensions that reflect sustainable employability include a range of concepts such as competence, affect, motivation, and specific behaviours [19]. Nauta *et al.* [20] have proposed at least four clusters for an employee’s working capacity that are essential to obtain and maintain sustainable employability: *health*, *involvement*, *motivation,* and *competence*. Since sustainable employability is a multi-factorial concept, instruments to determine possible threats or strengths to achieve a good balance should address all these personal and work-related factors.

Unfortunately, few instruments are available to assess these aspects in a feasible and scientifically reliable and valid way. Existing diagnostic instruments that measure health, work engagement, and ability are often extensive, have limited accessibility, and are one-dimensional. Scientifically validated instruments are seldom applied in practice [21]. Employees and employers could benefit from well-designed instruments for sustainable employability that are relatively easy to apply in their daily work schedule. In summary, to address the aforementioned issues, research instruments should use an integrative approach, be evidence-based *and* user-friendly [22].

In order to meet these requirements a ‘Vitality Scan’ was developed. Initially, a list of signs of possible stagnation for educational professionals was constructed. The list was subsequently modified into a more generic list for Dutch workers [23]. The current Vitality Scan is based on theoretical models and expert knowledge regarding person-job fit, work ability, and worker’s health, which are all brought together under four main categories, *health*, *involvement*, *motivation,* and *competence* [20].

The objective of the present study was to assess the internal consistency and construct validity of the Vitality Scan. For testing the construct validity, a priori hypotheses were defined for expected statistically significant differences between known groups [24].

*Hypothesis 1:* We expect older workers to report more problems (signs of stagnation) than younger workers, since age has been found to be negatively associated with physical health status and career perspective [21, 25, 26].

*Hypothesis 2:* We expect lower educated workers to report more problems (signs of stagnation) than higher educated workers since education level has been found to be negatively associated with exposure to several work-related risk factors and sickness absence [27].

## Methods

### Sample

Employees throughout the Netherlands who were working more than 16 h per week were online invited to fill out a questionnaire between February 2011 and July 2011. Members of an online panel on employability issues were included in the invitation.

In the Netherlands, in 2011, 80 % of the workforce worked 20 h or more per week and 10.8 % worked less than 12 h a week [28]. Since the purpose of the Vitality Scan was to explore risk factors that threaten sustainable employability in the working environment, we chose to include respondents who worked at least 16 h a week. Otherwise, signs of stagnation could just as easily be caused by non-work-related factors (personal, family or other), which is not the focus of this study. The actual online distribution of the scan was performed by an agency specialized in online surveys. Initially, 2707 respondents accepted the invitation and registered to complete the questionnaire. After the online distribution period ended, the dataset was cleaned by deleting empty files (N = 872 representing 37.6 % unemployed respondents, 48.1 % respondents working less than 16 h/week, and 14.3 % respondents working 16 h or more) and the file of one respondent working less than 16 h/week, which resulted in a sample of 1834 workers who participated in this study.

From this sample, participants were excluded if:

- More than 10 % (*i.e.* >3) of the answers on 31 items were missing (N = 112), or
- Age was <18 or > 65 years.

The raw dataset showed a clear distinction in various ‘types’ of missing data: respondents who did not actually start the questionnaire and respondents who did start, but stopped filling in the questionnaire after completing a small number of items. Each of these survey results was removed from the dataset. Of the remaining group (N = 1722), 36 respondents randomly missed an answer, but not more than three items (=10 %). We deemed these questionnaires as worthwhile and they remained in the dataset for analyses. Age groups were divided into three subgroups: younger workers (18–34 years), middle-aged workers (35–54 years), and older workers (55–65 years). Education level was defined according to the International Standard Classification of Education (ISCED-97) which led to the groups ‘lower educated’ , ‘mid-level educated’ , and ‘higher educated’. All respondents had a work contract of > 16 h per week.

### Construction of the vitality scan

To measure potential signs of stagnation for each of the four theoretical constructs as recommended [20], four (competence) or nine items (health, involvement, motivation) for each construct were formulated based on expert group discussions and theoretical review of determinants of employability. The items were formulated as statements and participants were asked to indicate to what extent they agreed with the statements. The items had a 5-point Likert rating scale ranging from (1) totally disagree to (5) totally agree. As stated, the Vitality Scan initially had four subscales. *Health* was considered to address both physical and mental well-being. Example items were ‘I feel energetic’ , ‘After a day’s work, I am out of energy’ , and ‘I have trouble relaxing’. *Involvement* referred to a sense of togetherness with the organisation and colleagues; it was about feeling valued. Example items were, ‘I can count on my colleagues when necessary’ , ‘I react curtly to others more often’ , and ‘I am proud of the organisation I work at’. *Motivatio*n was about enjoying work, a willingness to perform, and work satisfaction. Example items included: ‘After my holidays ended, I looked forward to going back to work’ , ‘I am less motivated than before’ , and ‘I question the purpose of my job’. Finally, the *competence* scale consisted of four items about having the knowledge and skills to perform in one’s current and future jobs. Example items included: ‘I work harder to accomplish the same’ and ‘I am having more difficulties in decision making than before’. In total, nine items were positively phrased and the answers to these questions were recoded before analysis. The original version of the Vitality Scan was in Dutch. The English version as presented in this article is a translation; neither back-translation nor pilot-testing in English has been done yet. Total possible scores ranged from 31–155. Participants were also asked about their age, gender, level of education, and total hours of employment.

### Statistical analyses

Analyses were conducted using SPSS statistics version 22 for MAC. Descriptive analyses (means, standard deviations) were performed first. The level of statistical significance was set at *p* < 0.05.

### Internal consistency

Internal consistency was calculated by examining the Cronbach’s alpha coefficient for the subscales and the total list of items. This was done twice, first, using items in the subscales according to the initial allocation [20] and second, items in the subscales according to subsequent factor analysis. In this study, a good Cronbach’s coefficient alpha was established as > 0.80 in accordance with the guideline proposed by Iacobucci & Duhachek [29], who concluded that the often-used > 0.70 may provide weak evidence, especially when applied to diagnostic instrument development.

### Construct validity

The Vitality Scan’s structure was examined by factor analysis using a principal component extraction method with varimax rotation for each conceptual group of items. Items were considered to be indicators of the same concept if they were highly related to each other by having factor loadings higher than 0.40.

Multiple group comparisons were performed to test the hypotheses that had been defined a priori about expected significantly distinct scores on known subgroups using an ANOVA.

### Ethical considerations

Survey participants were informed about the study and since they decided whether or not to complete the online questionnaire, written informed consent was not obtained. The study was exempt from review by a Medical Research Ethics Committee in accordance with the local regulatory guidelines and standards for human subjects protection in the Netherlands (Medical Research Involving Human Subjects Act) (WMO, 2005). Privacy was secured since the dataset was anonymous and the research team did not know who the respondents were.

## Results

### Sample characteristics

The final sample of 1722 respondents consisted of 746 women (43.3 %) and 961 men (55.8 %); 15 respondents did not answer this question (0.9 %). Ages ranged from 18–65 years (Mean = 45.7; SD = 12.5). Table 1 displays demographic characteristics and total stagnation scores initially (31 items) and following the reduction of items after analyses (28 items).

**Table 1:** Demographic characteristics and total stagnation scores initially (31 items) and following the reduction of items after analyses (28 items)

	%	N	31 items list mean scores (range; SD)	28 items list mean scores (range; SD)	N
*Age* (yrs) (mean 45.7; SD 12.5)					
Young (18–34)	21.9	377	106.5 (54–147; 16.9)	95.9 (41–132; 16.0)	373
Middle aged (35–54)	45.3	780	108.3 (44–152; 18.1)	97.6 (37–139; 17.3)	763
Older (55–65)	28.4	489	111.9 (50–155; 16.7)	101 (50–140; 16.0)	477
Missing	4.4	76			109
*Gender*					
Male	55.8	961	109.0 (50–153; 17.2)	98.3 (38–138; 16.4)	947
Female	43.3	746	108.5 (44–155; 18.3)	97.6 (37–140; 17.4)	724
Missing	0.9	15			51
*Level of education*					
Lower educated	11.1	192	107.6 (52–146; 19.9)	97.3 (41–138; 18.9)	186
Midlevel educated	52.0	896	109.0 (44–155; 17.7)	98.3 (37–140; 16.8)	880
Higher educated	36.2	623	108.8 (50–153; 16.9)	97.9 (38–138; 16.2)	610
Missing	0.6	11			46
*Group score*			108.8 (44–155; 17.7)	98.1 (37–140; 16.8)	1686
Missing					36
*Overall*		1722			1722

### Internal consistency

The internal consistency of the subscales according to Nauta *et al.* [20] and the total set of items on the overall scale were acceptable to good. Alphas were as follows: overall scale α = 0.94, health subscale α = 0.85, involvement subscale α =0.79, motivation subscale α = 0.84, and competence subscale α = 0.73. Results showed that the subscales for health, involvement and motivation would have had an increased Cronbach’s alpha if one item was deleted; however, this increase was less than 0.05. Inter-item correlations ranged widely within all four subscales.

### Construct validity

The results of the factor analysis are presented in Table 2.

**Table 2:** Rotated component matrix of the Vitality Scan in relation to the four clusters [20]

Item	Component^a^					
	1	2	3	4	5	6
*Health*						
I feel fit				**.722**		
I have trouble relaxing	.422			**.583**		
I worry more about things regarding work		**.502**				
I feel overloaded by my work	**.463**	**.463**		.456		
I called in sick more often this past year^b^					**.598**	
I sleep well				**.703**		
I am more annoyed by things that don’t go well at work		**.709**				
I am more emotional than before				**.493**		
After a day’s work, I am out of energy				**.523**		
*Involvement*						
I have less time to spend on hobbies, family and friends	**.557**			.426		
I feel involved with my colleagues						**.776**
I can count on colleagues when necessary						**.785**
I am more cynical about the organization than before		**.689**				
I only accept tasks within my job description^b^					**.563**	
I am proud of the organization I work at			**.601**			
I feel harassed quickly		**.424**				
I have lost interest in my job		.432	.**607**			
I react curtly to others more often		**.439**				
*Motivation*						
I am not succeeding in keeping up with my profession lately	**.734**					
I am less motivated than before	.427	.454	**.535**			
I have more trouble in focussing at work	**.661**					
I am not succeeding in keeping my appointments lately	**.681**					
I love my profession			**.732**			
I question the purpose of my job			**.434**			
When working, I forget about the time^c^			**.571**			
After the holidays, I looked forward going back to work			**.711**			
Everyday, I enjoy going to work			**.698**			
*Competence*						
I work harder than before to accomplish the same	**.594**					
I am less involved in chores with colleagues than before	**.526**					
I am having more difficulties in decision making than before	**.686**					
I am in dispute with my supervisor more often than before		**.632**				

Factor loadings are presented when higher than 0.40, and the highest loadings per item are in bold

^a^Component 1 = new subscale *Balance and Competence*; component 2 = new subscale *Resilience*; component 3 = new subscale *Motivation and Involvement*; component 4 = new subscale *Mental and Physical Health*; component 6 = *new subscale Social Support at work*

^b^Items were deleted because of negative contributions to internal consistency (initial calculations) and non-related content

^c^Item was deleted after internal consistency calculations on new subscales because of a negative contribution

After factor analysis and an examination of internal consistency, five subscales were constructed. Two items were deleted because of their negative contribution to the internal consistency and divergent content; ultimately, 29 items remained. Again, Cronbach’s alpha was used to establish reliability. The alpha coefficient on the new total set of items remained good (0.94). The first new subscale, *balance and competence* (N items = 8; α = 0.87), addressed aspects of work-life balance and feeling competent at work and included three of the four questions from the initial subscale on *competence*. The second new subscale reflected the degree of employee *resilience* (N items = 6; α = 0.84); there was no corresponding initial subscale. The third new subscale combined *motivation and involvement* issues (N items = 8; α = 0.86) and 8/18 questions from the initial subscales for *motivation* and *involvement* were included. The fourth *new* subscale addressed signs of *mental and physical health* (N items = 5; α = 0.80) and 5/9 items from the initial subscale *health* were included. Finally, the fifth *new* subscale* (component 6) *was about *social support at work* (N items = 2; α = 0.72) and both items were from the initial subscale for *involvement*. After further analysis of the new subscales’ reliability, another item was removed from the total set (and from the new subscale motivation and involvement) to increase internal consistency (Table 2), which resulted in a remaining total of 28 items.

### Difference between groups

ANOVA for group comparison showed a statistically significant difference in the overall Vitality Scan scores (N items = 28) between younger (N = 373), middle-aged (N = 763), and older (N = 477) workers (F [2,1610] = 10.778; *p* < 0.001). In accordance with our a priori hypothesis 1, the older workers reported the most problems (mean 101; SD 16.0), while younger workers reported the least problems (mean 95.9; SD 16.0). No statistically significant differences were found for education; therefore, hypothesis 2 was not confirmed.

## Discussion

This study describes the evaluation of the internal consistency and construct validity of a newly created Vitality Scan. The purpose of the Vitality Scan is to detect signs of stagnation in functioning as a measure of a worker’s sustainable employability. Following the analysis, the original 31 items were reduced to 28 items in the total set. The following items were removed (Table 2) because of a negative contribution to the internal consistency and divergent content: ‘I called in sick more often this past year’ , ‘I only accept tasks within my job description’, and ‘When working, I forget about the time’.

Internal consistency results, together with the underlying theoretical insights from a previous literature study and expert panels, provided evidence for a reliable and internally valid instrument [23]. Factor analysis extracted five factors. Cronbach’s alpha coefficients ranged from 0.72 (acceptable) to 0.87 (good) for the five factors. These factors represent signs on *balance and competence* (eight items), *motivation and involvement* (eight items), *resilience* (six items), *mental and physical health* (five items), and *social support at work* (two items). The definition of sustainable employability as proposed by Van der Klink *et al.* [8] contains similar components that reflect competences and attitudes, and motivational, health-related, contextual, and future-oriented factors. Additional literature also shows that the five distinct clusters found in this study each are essential contributors to a worker’s sustainable employability *e.g.* [30–33]. *Balance and competence* is about being able to achieve a balance in work and non-work demands and roles [10]. Such a balance affects a worker’s health and performance in a positive way [30]. *Motivation and commitment* is about having a positive attitude towards and being motivated to invest in one’s own competence development and working conditions. This resembles what Bakker and colleagues refer to as engagement [31]. *Resilience* refers to the ability to adapt to changing circumstances in job demands and organisational changes, which are a given in today’s work [9, 32]. Resilience facilitates adaptability, competence, and flexibility regardless of specific current or future circumstances [9, 10]. The *mental and physical health* items in the Vitality Scan, for example ‘I feel fit’ , resemble what Ryan and Frederick called ‘subjective vitality’ (a positive feeling of aliveness and energy) that covaries with psychological and somatic factors to impact the amount of available energy [33]. *Social support* resources in the organization are well known to be beneficial to a worker’s employability [30, 34]. Support from managers and colleagues plays in many ways a crucial (mediating) role with regard to the working capacity of the employees [35, 36].

In short, health, competences and motivation form the basic ‘floors’ of Ilmarinen’s House of Work Ability, which suggests that whereas the concept of work ability is the result of an interaction between employees’ capacities and characteristics together with work and environmental characteristics, the first three floors represent the necessary *conditions* for work ability and are employee-related [37]. We consider the aforementioned findings generally supportive to the content validity of the newly created Vitality Scan. The initial structure of the list of items in the Vitality Scan, which included constructs of health, involvement, motivation, and competence as suggested by Nauta *et al.* [20], was not fully confirmed, although the alphas were acceptable to good. However, further analysis showed a different than expected structure within the items of the Vitality Scan and this structure can be supported by existing literature. The high (0.94) alpha coefficient for the overall set of items may indicate that the Vitality Scan addresses one generic construct, *i.e.* sustainable employability, rather than five distinct underlying components. It has been suggested that sustainable employability is in fact a multifactorial concept and “to understand the implications of any of the given factors and of employability as a *gestalt*, one must examine the entire constellation of factors” [9].

Based on reports of working conditions in the Netherlands [25, 27], we expected to find differences in total scores for various age groups and levels of education. Older workers indeed showed significantly higher scores, indicating more possible problems in their employability. This is in line with our expectation and supports confidence on the construct validity of the Vitality Scan. However, research has also shown mixed evidence of the impact of chronological age on employability. Some reduction of physical and mental capability is inevitable, but this does not appear to be a reliable predictor of actual employability [1, 38, 39].

The expected difference in scores among levels of education was not found. This may be because although lower educated workers experience more frequent exposure to several work-related risk factors and sickness absences than higher educated workers, this is counter-balanced by a higher work-related psychosocial workload reported by the higher educated workers [27]. Unfortunately, we have no further information on the specific work-related risk factors for the participants in this study. Information on the participants’ professions or actual working hours was not obtained.

### Strengths and limitations of the study

The current study included a sample of 1722 workers. Distribution of gender was comparable to the Dutch working population as a whole. However, the sample in this study proved to be somewhat older and somewhat higher educated [28]. Internal consistency measures showed good reliability overall. Despite the promising results on internal consistency, user-friendliness, and content validity, there were also limitations to this study.

First, criterion validity [24] could not be established, because no existing scale of sustainable employability or underlying constructs was included in this study. Second, item formulation may have been ambiguous. Pilot testing on the comprehensibility of the items might have improved content validity [24, 40].

Third, a part of the respondents (the exact number is not known) who were invited to fill out the questionnaire are members of a panel group that regularly joins surveys and discussions on employability issues. These participants may have been more eager to share their experiences because they had already encountered difficulties in their work or with sustainable employability. Therefore, the scores found in the sample may have been biased and can differ from those in the general Dutch working population, thus hampering generalisation. No information on the respondents’ occupations and working conditions was available. Age distribution showed a slightly older sample than the Dutch working population. However, literature on age and sustainable employability is mixed and shows no clear direction for the implications, as we mentioned earlier. The limited information on the characteristics of the participants’ work environment (job characteristics, working hours, profession) restricts the representativeness of the results.

### Implications for further research and for practice

Follow-up studies should measure criterion validity [24] of the Vitality Scan by comparing scores to those of a commonly used measurement instrument on sustainable employability. Inter-item correlates should also be examined to indicate the discriminant value of the proposed five factors. Sustainable employability is not only a multidimensional concept, but also a dynamic one. This means that a worker’s employability can change over time [8, 9]. More information about specific working conditions and professions should be incorporated in the survey to enhance the representativeness of the outcomes.

The newly created Vitality Scan serves as a practical and theory-based instrument to give insight into employability over time. Employees and employers can discuss the outcomes of the Vitality Scan in light of career development and staying vital and healthy when working. Recent research emphasizes the importance of employee and employer dialogue about the various aspects of sustainable employability as a key to job satisfaction, job retention, and the long-term possibility of staying employable [18]. Van Vuuren [41] stated that interventions should not only be used for cure in cases of employability stagnation, but also for prevention and amplition purposes. The newly created Vitality Scan provides a quick evaluation of the current sustainable employability ‘status’ of the employee. We recommend that the Vitality Scan will be further developed into two separate lists with similar content: one version for the employee and one version for the supervisor. Combining these two perspectives (employee self-reports and supervisor ratings) could contribute to the informative value of the measurement [10]. Outcomes can serve as a practical dialogue guide on specific issues to be addressed. Contextual, work-related and non-work-related, factors can be discussed along with the outcomes of the Vitality Scan. Employees can use this instrument to take charge of improving or perpetuating their sustainable employability by detecting specific areas for career intervention [9, 10]. Communication between employees and their supervisors along with experienced supervisor support might influence determinants of work ability, work stress and job satisfaction, and therefore, also sustainable employability [42, 43]. The Vitality Scan serves in this stage as an evaluative monitoring instrument and is now valid only for this purpose. No selection or discriminative decisions on employees’ job options should be made based on the employee’s score.

The Vitality Scan could have increased value if a manual was created to explain the extent to which the individual scores indicate the specific strengths and problems of an employee’s sustainable employability, including a differentiation of the underlying five factors.

## Conclusions

This study showed that a newly developed Vitality Scan with 28 items had good internal consistency and promising content validity. The Vitality Scan demonstrated good properties as an evaluative instrument on workers’ sustainable employability. The Vitality Scan will increase in its value when predictive and discriminative possibilities become determined. To indicate the actual severity of reported signs of employee functional stagnation, more research is needed.
